# Protein Disulfide Isomerase Involvement in Dilated Cardiomyopathy Caused by Filamin C Deficiency in Male Mice

**DOI:** 10.1111/jcmm.70493

**Published:** 2025-03-18

**Authors:** He Xuan, Chenghao Fan, Xue Bai, Anteng Shi, Yu Nie, Shengshou Hu, Hong Lian

**Affiliations:** ^1^ State Key Laboratory of Cardiovascular Disease, Fuwai Hospital, National Center for Cardiovascular Disease Chinese Academy of Medical Sciences and Peking Union Medical College Beijing China; ^2^ Beijing Key Laboratory of Preclinical Research and Evaluation for Cardiovascular Implant Materials, Animal Experimental Center, Fuwai Hospital, National Center for Cardiovascular Disease Chinese Academy of Medical Sciences and Peking Union Medical College Beijing China

**Keywords:** dilated cardiomyopathy, E64FC26, FLNC, PDI

## Abstract

Loss‐of‐function variants in the *FLNC* gene, which encodes Filamin C, cause dilated cardiomyopathy with a high risk of life‐threatening arrhythmias. Therapies targeting the underlying mechanism of *FLNC*‐related dilated cardiomyopathy remain limited. In this study, we observed that deletion of *Flnc* in cardiomyocytes of mice led to prominent ventricular dilation, cardiac dysfunction, and cardiac fibrosis. This phenotype closely resembles *FLNC*‐related dilated cardiomyopathy in humans. RNA sequencing analysis revealed activation of protein disulfide isomerase (PDI) in *Flnc*‐deleted cardiac tissues, as confirmed by immunoblotting. Treatment with the specific PDI inhibitor E64FC26 improved cardiac function, reduced cardiac fibrosis, and decreased cardiomyocyte apoptosis in cardiomyocyte‐specific *Flnc*‐deleted mice. We provide evidence that PDI is involved in the cardiac remodeling induced by Filamin C deficiency, and that treatment with the PDI inhibitor resulted in beneficial effects in mice with dilated cardiomyopathy caused by *Flnc* deletion. Our findings suggest that PDI could be a promising therapeutic target for *FLNC*‐related dilated cardiomyopathy.

## 1 Introduction

Dilated cardiomyopathy (DCM) is an important cause of heart failure [1]. Estimates suggest that pathogenic variants can be identified in approximately 20% of sporadic DCM patients and around 30% of familial DCM patients [1]. *Filamin C* (*FLNC*) was identified as a pathogenic gene for DCM, associated with a high risk of sudden death [2]. Our previous work found the prevalence of *FLNC* variants in the Chinese DCM population was significantly high (15.4%), and patients with *FLNC* variants exhibited more severe clinical symptoms [3]. The aim of this study is to explore therapeutic strategies targeting the underlying molecular mechanisms associated with *FLNC*‐related DCM.

Endoplasmic reticulum (ER) stress, characterised by impaired proteostasis due to the accumulation of unfolded or misfolded proteins, is associated with adverse clinical outcomes in DCM patients, as evidenced by its activation in the myocardial tissues of patients at the heart failure stage [4, 5]. Protein disulfide isomerases (PDI) are essential protein folding chaperones that play crucial roles in regulating various aspects of ER stress. Additionally, they are involved in the pathophysiological processes of cardiovascular diseases such as thrombosis, stroke, and myocardial infarction [6]. However, there is no evidence on whether PDI plays a role in the progression of *FLNC*‐related DCM or if it could serve as a potential therapeutic target.

## 2 Results

### 2.1 *Flnc^iKO^* mice exhibited the dilated cardiomyopathy phenotype with PDI activation

**FIGURE 1:**
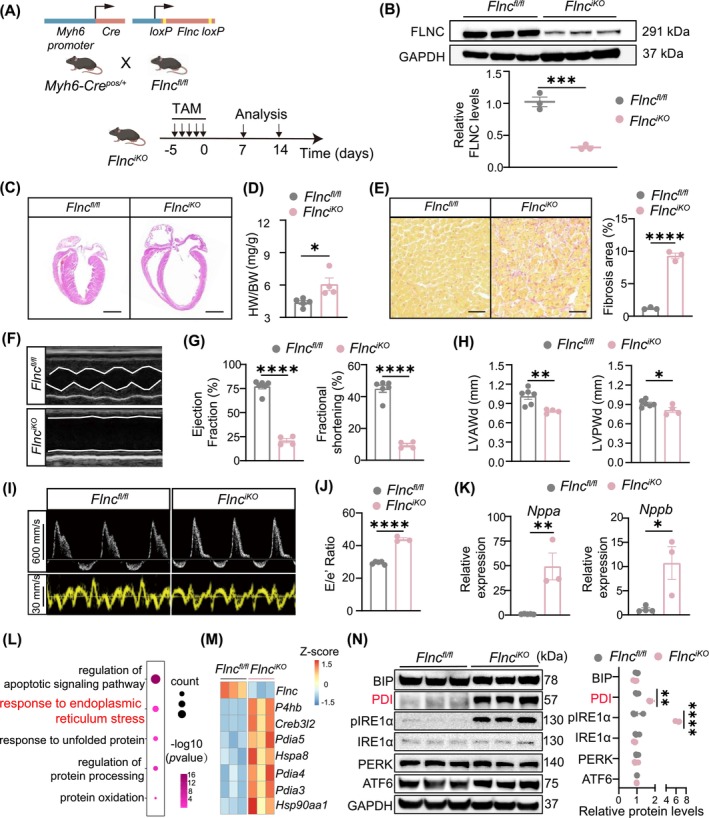
*Flnc* ^*iKO*^ mice presented the malignant dilated cardiomyopathy phenotype. (A) Schematic of *Flnc* ^*iKO*^ mouse generation. (B) Western blot and quantification of Filamin C levels in *Flnc* ^*iKO*^ mice hearts 7 days after tamoxifen treatment (20 mg/kg/d for 5 days). *Flnc* ^*fl/fl*^ mouse hearts served as controls (*n* = 3, each). (C) Haematoxylin & Eosin‐stained heart sections from *Flnc* ^*iKO*^ mice and control *Flnc* ^*fl/fl*^ mice. Scale bar, 2000 μm. (D) Heart weight/body weight ratios (HW/BW) of *Flnc* ^*iKO*^ mice and *Flnc* ^*fl/fl*^ mice 2 weeks after tamoxifen treatment (*n* = 5, each). (E) Images and quantification of cardiac fibrosis in Sirius Red‐stained sections from *Flnc* ^*iKO*^ mice and *Flnc* ^*fl/fl*^ controls (*n* = 3, each). Scale bar, 50 μm. (F–H) Representative echocardiography images and quantification of parameters, including ejection fraction, fractional shortening, left ventricular end‐diastolic anterior wall thickness (LVAWd) and the left ventricular end‐diastolic posterior wall thickness (LVPWd) of *Flnc* ^*iKO*^ mice (*n* = 4) and control *Flnc* ^*fl/fl*^ mice (*n* = 6) 2 weeks after tamoxifen treatment. (I, J) Representative images of echocardiography and quantification analysis of E/e' ratio of *Flnc* ^*iKO*^ mice and control *Flnc* ^*fl/fl*^ mice 2 weeks after tamoxifen treatment (*n* = 3, each). (K) RT‐qPCR analysis of *Nppa* and *Nppb* expression in *Flnc* ^*iKO*^ mice hearts (*n* = 3, each) compared to *Flnc* ^*fl/fl*^ mice (*n* = 5 for *Nppa* and *n* = 4 for *Nppb*). (L) Dotplot illustrating selected GO terms from a gene ontology analysis of differentially expressed genes (DEGs) identified through RNA sequencing. The colour of each dot represents the *p* value, while the size of each dot is proportional to the number of DEGs within that category. (M) Heatmap of DEG representing endoplasmic reticulum stress associated transcripts. (N) Western blot analysis and quantification for ER stress and UPR associated proteins from *Flnc* ^*iKO*^ mice and *Flnc* ^*fl/fl*^ controls hearts (*n* = 3, each). Data are mean ± SEM. Two group comparison was applicated using two‐tailed Student's *t*‐test. Multiple comparisons were made using one‐way ANOVA. *p < 0.05, **p < 0.01, ***p < 0.001, and ****p < 0.0001.

To mimic *FLNC*‐related DCM, we used a tamoxifen‐inducible Cre‐mediated recombination system to delete *FLNC* in cardiomyocytes. Homozygous *Flnc*‐floxed mice (*Flnc* ^*fl/fl*^) were crossed with *Myh6‐MerCreMer* transgenic mice to generate *Flnc* ^*iKO*^ mice (Figure 1A). FLNC protein was efficiently deleted in the hearts of *Flnc* ^*iKO*^ mice but not *Flnc* ^*fl/fl*^ control mice 7 days after tamoxifen treatment (Figure 1B). Two weeks after tamoxifen administration, the *Flnc* ^*iKO*^ mice exhibited significant cardiac dilation with increased heart mass (Figure 1C,D). *Flnc* ^*iKO*^ mice also displayed substantial cardiac fibrosis (Figure 1E), consistent with the clinical presentation of interstitial fibrosis in *FLNC*‐related DCM [7]. Echocardiographic results showed a swift decline in ejection fraction and fractional shortening, along with reduced left ventricular end‐diastolic thickness and an increased E/e' ratio in *Flnc* ^*iKO*^ mice (Figure 1F–J), demonstrating impaired cardiac function. RT‐qPCR analysis revealed that levels of *Nppa* and *Nppb*, the heart failure biomarkers, dramatically increased in *Flnc* ^*iKO*^ mouse hearts (Figure 1K). In summary, deletion of FLNC in adult cardiomyocytes led to severe DCM characterised by adverse cardiac remodelling and heart failure.

To further explore the molecular mechanisms of *FLNC*‐related DCM, cardiac ventricles of *Flnc* ^*iKO*^ mice after 2 weeks of tamoxifen treatment were subjected to RNA sequencing. Gene ontology analysis revealed enrichment in ER stress and unfolded protein response pathways (Figure 1L). ER stress‐associated genes were upregulated in *Flnc* ^*iKO*^ mouse hearts compared to controls (Figure 1M). Members of PDI family, which coded the protein folding chaperones induced by protein misfolding and played crucial roles in ER stress, were significantly activated (Figure 1M). Western blot analysis confirmed increased PDI protein level in the *Flnc* ^*iKO*^ mouse hearts (Figure 1N). Phosphorylated IRE1a also upregulated, while the levels of other ER stress‐associated proteins remained stable (Figure 1N). These results demonstrated the activation of ER stress in hearts of *Flnc* ^*iKO*^ mouse and suggested PDI may be instrumental in the pathophysiology of DCM in these mice.

### 2.2 Inhibition of PDI alleviated cardiac dysfunction, fibrosis, and cardiomyocyte apoptosis in *Flnc^iKO^* mice

**FIGURE 2:**
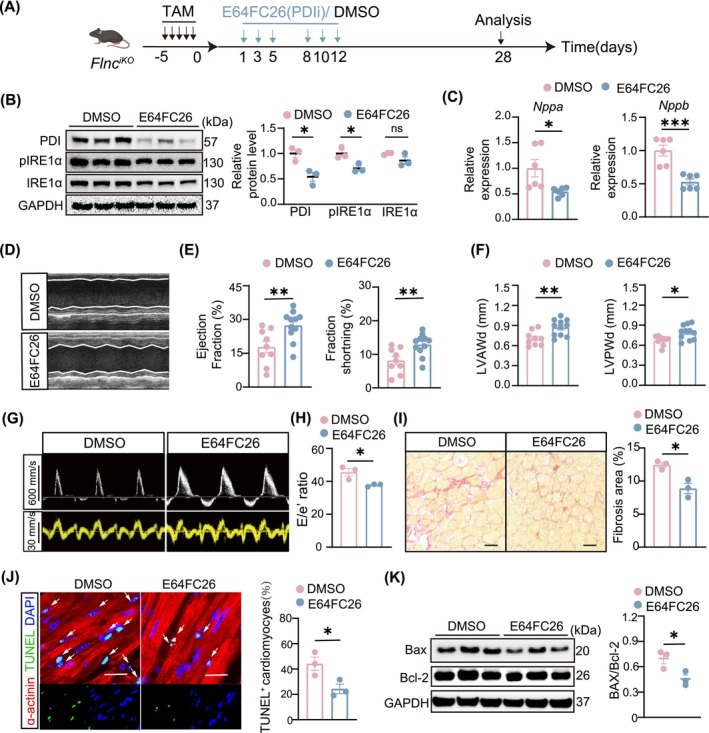
Inhibiting PDI with E64FC26 protected cardiac function and reduced cardiomyocyte apoptosis in *Flnc* ^*iKO*^ mice. (A) Schematic of experimental design. For *Flnc* ^*iKO*^ mice, E64FC26 (2 mg/kg) or vehicle DMSO was administered by intraperitoneal injection 3 times per week for 2 weeks. Hearts were harvested immediately after drug administration for PDI expression assay. Two weeks after administration completion, cardiac function, cardiac fibrosis and apoptosis were evaluated. (B) Western blot analysis and quantification of PDI, IRE1α, and phosphorylation of IRE1α levels in *Flnc* ^*iKO*^ mice, after E64FC26 or DMSO treatment (*n* = 3, each). (C) RT‐qPCR analysis of *Nppa* and *Nppb* expression in hearts of *Flnc* ^*iKO*^ mice treated with E64FC26 or DMSO (*n* = 6, each). (D) Representative echocardiography M‐mode images of *Flnc* ^*iKO*^ mice treated with E64FC26 or DMSO after 2 weeks of drug administration completion. (E, F) Echocardiographic measurements of ejection fraction, fractional shortening, left ventricular end‐diastolic anterior wall thickness (LVAWd) and the left ventricular end‐diastolic posterior wall thickness (LVPWd) of *Flnc* ^*iKO*^ mice treated with E64FC26 (*n* = 9) or DMSO (*n* = 11) after 2 weeks of drug administration completion. (G, H) Representative images of echocardiography and quantification analysis of E/e' ratio of *Flnc* ^*iKO*^ mice treated with E64FC26 or DMSO (*n* = 3, each). (I) Sirus‐Red staining and quantification of fibrosis areas on heart sections from *Flnc* ^*iKO*^ with E64FC26 (*n* = 3) or DMSO (*n* = 3) after drug administration. Scale bar, 50 μm. (J) Immunofluorescence staining images and quantification of relative TUNEL‐positive cardiomyocytes in *Flnc* ^*iKO*^ mouse hearts 2 weeks after treated with E64FC26 or DMSO (*n* = 3 per group). Scale bar, 20 μm. (K) Western blot analysis of apoptosis by detection of Bax and Bcl2 in *Flnc* ^*iKO*^ mouse hearts treated with E64FC26 or DMSO (*n* = 3 per group). Data are shown as mean ± SEM. Statistical analysis was performed using two‐tailed, unpaired Student's *t*‐tests for two groups comparison. For multiple comparisons, one‐way ANOVA was employed. Multiple comparisons were made using one‐way ANOVA. ns, not significant. *p < 0.05, **p < 0.01 and ***p < 0.001.

To evaluate whether PDI could serve as a potential therapeutic target in *FLNC*‐related DCM, we conducted a study in *Flnc* ^*iKO*^ mice utilising a pan‐PDI inhibitor (E64FC26). E64FC26 was administered to *Flnc* ^*iKO*^ mice via intraperitoneal injection at a dose of 2 mg/kg on Day 1,3, and 5 per week for a period of 14 days (Figure 2A). The expression levels of PDI and phosphorylation of IRE1α were significantly reduced (Figure 2B), indicating successful suppression of PDI and the arm of ER stress in the hearts of *Flnc* ^*iKO*^ mice. RT‐qPCR analysis revealed that the levels of *Nppa* and *Nppb* were attenuated in the hearts of *Flnc* ^*iKO*^ mice after E64FC26 administration (Figure 2C). Echocardiographic assessment demonstrated that E64FC26 treatment improved both systolic and diastolic cardiac function of *Flnc* ^*iKO*^ mice, as evidenced by increased ejection fraction and fractional shortening, alongside increased left ventricular end‐diastolic thickness and reduced E/e' ratio (Figure 2D–H). Picrosirius‐red staining showed a 30% reduction in cardiac fibrosis in E64FC26‐treated *Flnc* ^*iKO*^ mice compared to controls (Figure 2I). These results demonstrated that PDI inhibitor treatment alleviated the progression of heart failure in *Flnc* ^*iKO*^ mice with DCM. Cardiomyocyte apoptosis is a common pathophysiological process in DCM [8]. Apoptosis pathways were activated in the hearts of *Flnc* ^*iKO*^ mice (Figure 1L); we next asked whether E64FC26 treatment would regulate cardiomyocyte apoptosis. Terminal deoxynucleotidyl transferase dUTP nick end labeling (TUNEL) assays showed approximately a 50% reduction in apoptotic cardiomyocytes in *Flnc* ^*iKO*^ mice treated with E64FC26 compared to vehicle‐treated mice (Figure 2J). Western blot showed that E64FC26 treatment led to a reduced Bax to Bcl‐2 ratio, demonstrating that PDI inhibitor treatment mitigated cardiomyocyte apoptosis in *Flnc* ^*iKO*^ mice (Figure 2K).

## 3 Discussion

Heterozygous truncating *FLNC* variants are the predominant pathogenic mutation type observed in *FLNC*‐DCM patients [9]. With over 80 heterozygous truncating mutations in *FLNC* associated with dilated cardiomyopathy, simulating each variant individually presents a significant challenge. Additionally, heterozygous *Flnc* variants in mouse model do not exhibit overt striated muscle phenotypes, which limits their utility in elucidating the pathogenesis of dilated cardiomyopathy [10]. To address this, we generated an inducible cardiomyocyte‐specific *Flnc* knock out mice (*Flnc* ^*iKO*^ mice), in which the region between exons 9 and 13 of *Flnc* was deleted through Cre‐mediated recombination to model the general conditions of DCM caused by *FLNC* truncating variants. Notably, a similar conditional knockout mouse model has been developed by Ju Chen's group, but their research emphasises the function of *FLNC* in both developing and adult cardiomyocytes [11]. Nevertheless, using the *Flnc* ^*iKO*^ mice, we observed a significant decrease in FLNC protein levels in the hearts, consistent with findings associated with *FLNC*‐DCM patients [2]. This model successfully recapitulated the malignant clinical manifestations of *FLNC‐*related DCM. Importantly, we identified potential therapeutic targets, specifically PDI, for *FLNC*‐related DCM using the *Flnc* ^*iKO*^ mice. These findings demonstrated that the *Flnc* ^*iKO*^ mouse can effectively replicate *FLNC*‐associated DCM in humans and serve as a model for exploring the mechanisms and potential targets related to *FLNC*‐DCM.

*FLNC* truncating variant not only directly produces misfolded FLNC protein but disrupts interactions with its various binding partners due to FLNC dysfunction [2, 10], both of which may lead to impairment of ER proteostasis. Through RNA‐seq and Western blot analysis, we identified the activation of ER stress and its component PDI in the hearts of *Flnc* ^*iKO*^ mice. Similar upregulation of PDI was observed in familial DCM caused by the truncating mutation in phospholamban (PLN) [12], suggesting the nonnegligible role of PDI in familial DCM. PDI has been applied as an intervention target for preventing skeletal muscle wasting resulting from cachexia, indicating its therapeutic potential in myopathy [13]. Additionally, inhibiting PDI with either an inhibitor or an antibody has demonstrated therapeutic effects in thromboinflammatory conditions and other cardiovascular diseases, supported by both basic research and clinical trials [14, 15]. These studies underscore the therapeutic value of targeting PDI and the pharmaceutical promise of PDI inhibitors for treating cardiovascular diseases. In our study, we treated *Flnc* ^*iKO*^ mice with the PDI inhibitor E64FC26, leading to alleviation of DCM phenotypes, as indicated by preserved cardiac function, reduced cardiac fibrosis areas, and decreased cardiomyocyte apoptosis.

In this study, we used *FLNC* ^*fl/fl*^ mice without the DCM phenotype as controls. Future studies could compare PDI activation in mice with DCM induced by different factors, such as acquired and genetic causes (including *FLNC* and other gene variants), using larger sample sizes to deepen the understanding of PDI's role in *FLNC*‐related DCM and its therapeutic potential.

Overall, we demonstrated for the first time that PDI is involved in cardiac remodelling in *Flnc* ^*iKO*^ mice, underscoring the translational value of PDI as a potential therapeutic target in *FLNC‐*related DCM.

## Author Contributions

**He Xuan:** data curation (equal), formal analysis (equal), project administration (equal), visualization (equal), writing – original draft (equal). **Chenghao Fan:** data curation (equal), formal analysis (equal), project administration (equal), visualization (equal), writing – original draft (equal). **Xue Bai:** data curation (supporting), visualization (supporting). **Anteng Shi:** methodology (supporting), resources (supporting). **Yu Nie:** supervision (supporting), writing – review and editing (equal). **Shengshou Hu:** conceptualization (equal), funding acquisition (lead), supervision (equal), writing – review and editing (supporting). **Hong Lian:** conceptualization (equal), supervision (equal), writing – review and editing (equal).

## Ethics Statement

All animal experiments were performed following the guidelines outlined in the Guide for the Use and Care of Laboratory Animals. All animal procedures in this work were approved by the Institutional Animal Care and Use Committee (IACUC) of Fuwai Hospital, Chinese Academy of Medical Sciences (Approval Number: FW‐2021‐0006).

## Conflicts of Interest

The authors declare no conflicts of interest.

## Data Availability

The data in the current study are available from the corresponding author on reasonable request.
